# Supplementary material for: Is the Morphological Subtype of Extra-Pulmonary Neuroendocrine Carcinoma Clinically Relevant?
Source: Cancers (Basel). 2021 Aug 18;13(16):4152. doi: 10.3390/cancers13164152 (PMC8392018; doi:10.3390/cancers13164152)
Supplement: Supplementary file 1 [file cancers-13-04152-s001.zip › cancers-1347306-supplementary.pdf]

Supplementary Materials

Is the morphological subtype of Extra-Pulmonary Neuroendocrine Carcinoma clinically relevant?

Melissa Frizziero [1], Alice Durand [2], Rodrigo G. Taboada [3], Elisa Zaninotto [4], Claudio Luchini [5], Bipasha Chakrabarty [6], Valérie Hervieu [7], Laura C. L. Claro [8], Cong Zhou [9], Sara Cingarlini [4], Michele Milella [4], Thomas Walter [2], Rachel S. Riechelmann [3], Angela Lamarca [10], Richard A. Hubner [10], Wasat Mansoor [10], Juan W. Valle [1] and Mairéad G. McNamara [1*]

Supplementary Table S1. Summary of treatment modalities for the 'potentially curable' subgroup.

	**Whole population**	**Small cell**	**Non-small cell**	**p-value**
	***n*=39**	***n*=13**	***n*=26**	
Surgery alone	12 (30.8%)	4 (30.8%)	8 (30.8%)	Surgery (+/- CTh or CTh+RTh) vs. CTh+RTh p=0.194
Surgery + CTh*	19 (48.7%)	5 (38.5%)	14 (53.8%)	
Surgery + CTh+RTh**	1 (2.6%)	0 (0.0%)	1 (3.8%)	
CTh+RTh	7 (17.9%)	4 (30.8%)	3 (11.5%)	Surgery + CTh vs. others p=0.320

**CT regimens applied in the curative setting**				
	***n*=27**	***n*=9**	***n*=18**	Platinum/Etoposide vs. others p=0.423<br>Fluorop/Oxa vs. others p=0.582
Platinum/Etoposide	18 (66.7%)	5 (55.6%)	13 (72.2%)	
Fluorop/Oxa	4 (14.8%)	2 (22.2%)	2 (11.1%)	
Platinum/Fluorop/Taxane	1 (3.7%)	1 (11.1%)	0 (0.0%)	
unknown	4 (14.8%)	1 (11.1%)	3 (16.7%)	

n = number of patients. CTh = chemotherapy. RTh = radiotherapy. Fluorop = fluoropyrimidine. Oxa = oxaliplatin. Platinum includes cisplatin or carboplatin. *adjuvant and/or neoadjuvant. **adjuvant. p values were determined by Fisher's exact test.

Supplementary Table S2. Summary of treatment modalities for the 'advanced' subgroup.

	**Whole population**	**SC**	**non-SC**	**p value**
**1st line management**	***n*=159**	***n*=73**	***n*=86**	
**Chemotherapy**	**148/159 (93.1%)**	**71/73 (97.3%)**	**77/86 (89.5%)**	CTh (+/- RTh or surgery) yes vs. no p=0.056
• CTh alone	137/148 (92.6%)	67/71 (94.4%)	70/77 (90.9%)	
• CTh + RTh	7/148 (4.7%)	3/71 (4.2%)	4/77 (5.2%)	
• CTh + surgery	4/148 (2.7%)	1/71 (1.4%)	3/77 (3.9%)	
• Platinum/Etoposide	118/148 (79.7%)	58/71 (81.7%)	60/77 (77.9%)	Platinum/Etoposide yes vs. no p=0.569<br>Fluorop +/-Iri +/-Oxa yes vs. no p=0.081
• Fluorop +/-Iri +/-Oxa	15/148 (10.1%)	4/71 (5.6%)	11/77 (14.3%)	
• Other platinum-based regimens	6/148 (4.1%)	3/71 (4.2%)	3/77 (3.9%)	
• Other regimens	9/148 (6.1%)	6/71 (8.4%)	3/77 (3.9%)	

**Somatostatin Analogue**	**1/159 (0.6%)**	**0/73 (0.0%)**	**1/86 (1.2%)**	
**Targeted therapy**	**1/159 (0.6%)**	**0/73 (0.0%)**	**1/86 (1.2%)**	
**Loco-regional treatment alone**	**6/159 (3.8%)**	**1/73 (1.4%)**	**5/86 (5.8%)**	
**BSC**	**3/159 (1.9%)**	**1/73 (1.4%)**	**2/86 (2.3%)**	
**2nd line management**	***n*=124**	***n*=58**	***n*=66**	
**Chemotherapy**	**83/124 (66.9%)**	**37/58 (63.8%)**	**46/66 (69.7%)**	CTh yes vs. no p=0.486
• CTh alone	83/83 (100.0%)	37/37 (100.0%)	46/46 (100.0%)	
• Fluorop/Iri	47/83 (56.6%)	20/37 (54.1%)	27/46 (58.7%)	Fluorop/Iri yes vs. no p=0.671 Platinum/Etoposide yes vs. no p=0.283
• Dacarbazine-based regimens	10/83 (12.0%)	4/37 (10.8%)	6/46 (13.0%)	
• Platinum/Etoposide	8/83 (9.6%)	5/37 (13.5%)	3/46 (6.5%)	
• Other regimens	18/83 (21.7%)	8/37 (21.6%)	10/46 (21.7%)	
**Somatostatin Analogue**	**1/124 (0.8%)**	**1/58 (1.7%)**	**0/66 (0.0%)**	
**Targeted therapy**	**4/124 (3.2%)**	**3/58 (5.2%)**	**1/66 (1.5%)**	
**Loco-regional treatment alone**	**5/124 (4.0%)**	**2/58 (3.4%)**	**3/66 (4.5%)**	
**PRRT**	**2/124 (1.6%)**	**1/58 (1.7%)**	**1/66 (1.5%)**	
**ICB**	**1/124 (0.8%)**	**0/58 (0.0%)**	**1/66 (1.5%)**	
**BSC/unknown**	**28/124 (22.6%)**	**14/58 (24.1%)**	**14/66 (21.2%)**	
**3rd line management**	***n*=80**	***n*=35**	***n*=45**	
**Chemotherapy**	**39/80 (48.8%)**	**17/35 (48.6%)**	**22/45 (48.9%)**	
• CTh alone	39/39 (100.0%)	17/17 (100%)	22/22 (100.0%)	
• Fluorop/Iri and/or Oxa	14/39 (35.9%)	6/17 (35.3%)	8/22 (36.4%)	
• Temozolomide-based regimens	9/39 (23.1%)	4/17 (28.6%)	5/22 (22.7%)	
• Dacarbazine-based regimens	7/39 (17.9%)	4/17 (28.6%)	3/22 (13.6%)	
• Other regimens	9/39 (23.1%)	3/17 (17.6%)	6/22 (27.3%)	
**Targeted therapy**	**8/80 (10.0%)**	**2/35 (5.7%)**	**6/45 (13.3%)**	

**ICB**	**4/80 (5.0%)**	**2/35 (5.7%)**	**2/45 (4.4%)**
**Interferon**	**1/80 (1.3%)**	**1/35 (2.9%)**	**0/45 (0.0%)**
**Loco-regional treatment alone**	**2/80 (2.5%)**	**1/35 (2.9%)**	**1/45 (2.2%)**
**BSC/unknown**	**26/80 (32.5%)**	**12/35 (34.3%)**	**14/45 (31.1%)**

n = number of patients. CTh = chemotherapy. RTh = radiotherapy. BSC = best supportive care. Fluorop = fluoropyrimidine. Iri = irinotecan. Oxa = oxaliplatin. Unknown = patient had died or was lost to follow-up or information on treatment is missing. ICB = immune checkpoint blockade. PRRT = peptide receptor radionuclide therapy. Loco-regional treatment alone include surgery or radiotherapy or the combination of the two. p value was determined by Pearson's chi-squared test.

Supplementary Table S3. Univariable and multivariable analysis for progression free survival and overall survival in two sub-populations of the advanced extra-pulmonary neuroendocrine carcinoma cohort.

		**Progression free survival**		**Overall survival**	
		**Univariable**	**Multivariable**	**Univariable**	**Multivariable**
		**HR, p value**	**HR, p value**	**HR, p value**	**HR, p value**
**CTh or CTh+RTh subgroup**		***n*=136**		***n*=143**	
Age at diagnosis#	Continuous	1.00, p=0.636		1.00, p=0.910	
Gender	Male vs. Female	0.84, p=0.353		0.77, p=0.191	
ECOG PS	≥2 vs. 0-1	2.65, p<0.005	1.97, p=0.007	2.72, p<0.005	2.29, p=0.001
Ki-67	≥55% vs. <55%	1.62, p=0.068	1.81, p=0.039	1.38, p=0.237	1.58, p=0.133
Site of origin	CUP vs. GEP	0.99, p=0.961		1.16, p=0.500	
	Others vs. GEP	0.88, p=0.798		0.86, p=0.775	
Morphological subtype	non-SC vs. SC	1.25, p=0.221	1.63, p=0.019	1.23, p=0.270	1.63, p=0.027
Interaction Ki-67/ Morphological subtype		0.48, p=0.274*		0.52, p=0.335*	
**Platinum/Etoposide subgroup**		***n*=109**		***n*=115**	
Age at diagnosis#	Continuous	1.00, p=0.842		1.00, p=0.632	
Gender	Male vs. Female	0.93, p=0.718		0.89, p=0.590	
ECOG PS	≥2 vs. 0-1	2.78, p<0.005	2.05, p=0.010	2.82, p<0.005	2.31, p=0.003
Ki-67	≥55% vs. <55%	1.92, p=0.066	2.06, p=0.055	1.91, p=0.075	0.66, p=0.577
Site of origin	CUP vs. GEP	1.02, p=0.922		1.14, p=0.578	
	Others vs. GEP	0.96, p=0.949		0.73, p=0.601	
Morphological subtype	non-SC vs. SC	1.22, p=0.326	1.53, p=0.065	1.14, p=0.539	0.364, p=0.228
Interaction Ki-67/ Morphological subtype		0.29, p=0.142*		0.20, p=0.036*	4.45, p=0.090

n = number of patients. HR = hazard ratio. 95%CI = 95%-confidence interval. ECOG PS = Eastern Cooperative Oncology Group performance status. GEP = gastro-entero-pancreatic tract. CUP = cancer of unknown primary. CTh = chemotherapy. RTh = radiotherapy. The ‘CTh or CTh+RTh subgroup’ includes all patients with advanced stage disease who received at least one line of palliative CTh or CTh+RTh. The ‘Platinum/Etoposide subgroup’ includes all patients with advanced stage disease who received platinum/etoposide chemotherapy in the first line setting. #at diagnosis of advanced stage disease. *interaction test by Cox-regression was significant and was therefore included in the multivariable analysis alongside the two individual variables and ECOG PS .

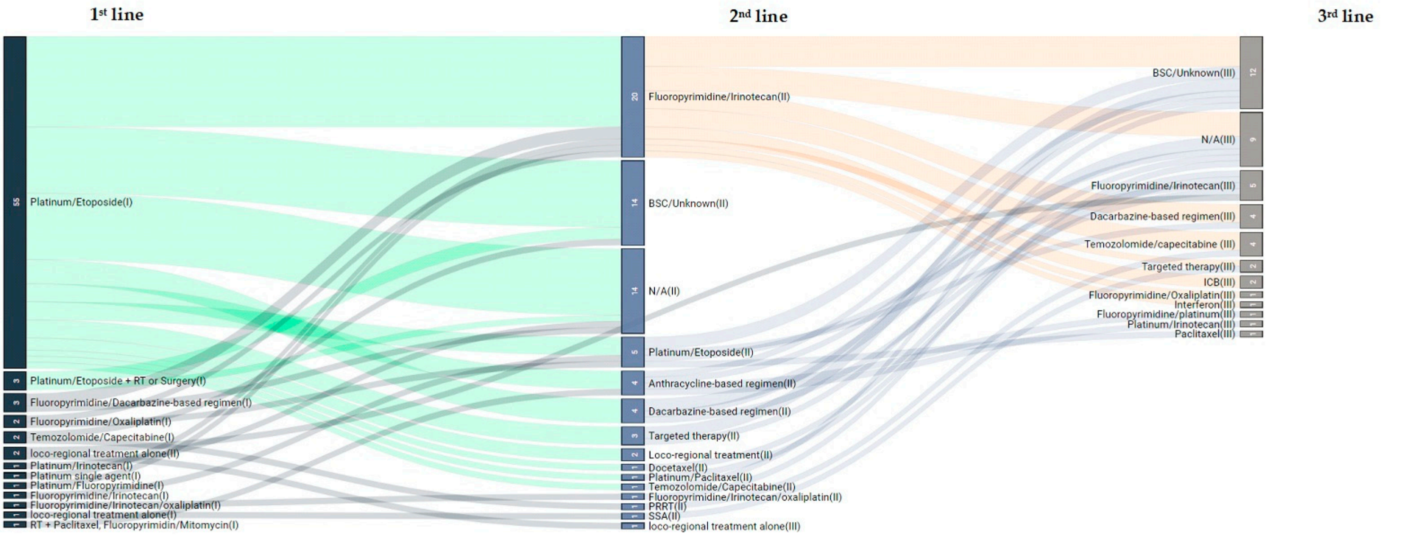

Supplementary Figure S1a. Sankey diagram of sequential treatment in patients with small cell EP-NEC. This shows the sequence of treatment strategies applied for each patient. The width of the lines is proportional to number of patients who received that specific treatment. RT = radiotherapy. PRRT = peptide receptor radionuclide therapy. SSA = somatostatin analogue. ICB = immune checkpoint blockade. Targeted therapy = sunitinib or everolimus. Loco-regional treatment alone = surgery or radiotherapy or the combination of the two. BSC = best supportive care. Unknown = patient had died or was lost to follow-up, or information on treatment is missing. N/A = not applicable; patient died during or soon after previous line of treatment or had not progressed at last follow-up.

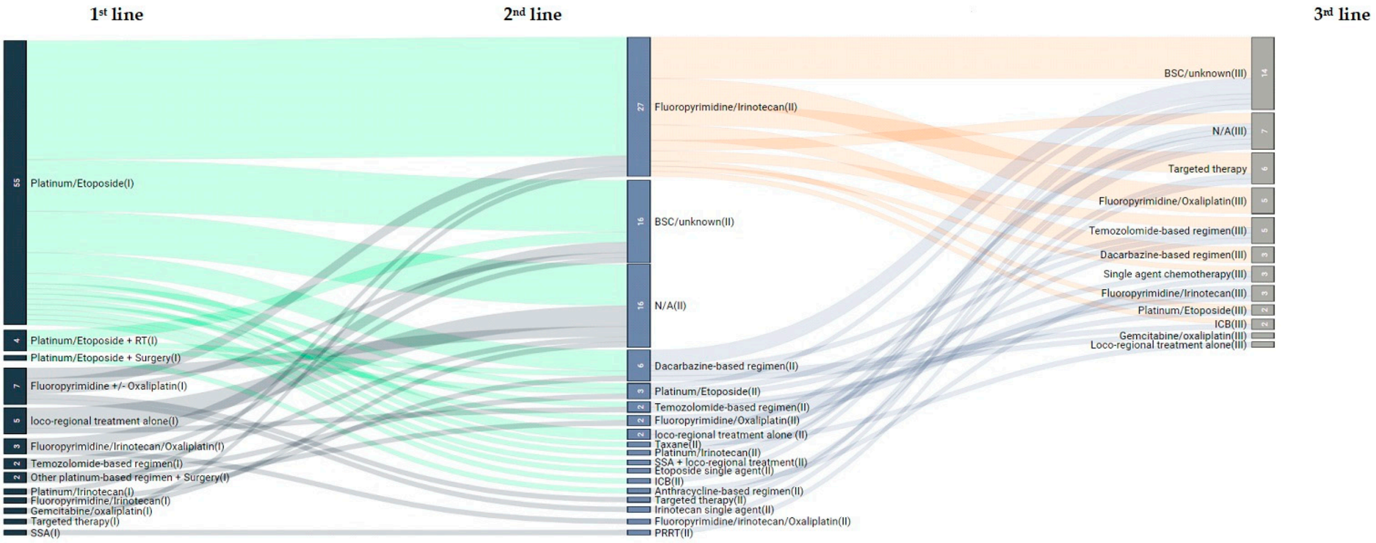

Supplementary Figure S1b. Sankey diagram of sequential treatment in patients with non-small cell EP-NEC. This shows the sequence of treatment strategies applied for each patient. The width of the lines is proportional to number of patients who received that specific treatment. RT = radiotherapy. PRRT = peptide receptor radionuclide therapy. SSA = somatostatin analogue. ICB = immune checkpoint blockade. Targeted therapy = sunitinib or everolimus. Loco-regional treatment alone = surgery or radiotherapy or the combination of the two. BSC = best supportive care. Unknown = patient had died or was lost to follow-up or information on treatment is missing. N/A = not applicable; patient died during or soon after previous line of treatment or had not progressed at last follow-up.

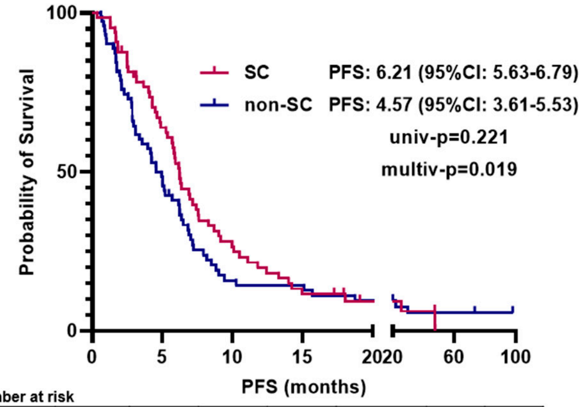

Number at risk

months	0	5	10	15	20	60	98.1
SC	65	40	16	7	3	0	0
non-SC	71	33	10	9	6	2	1

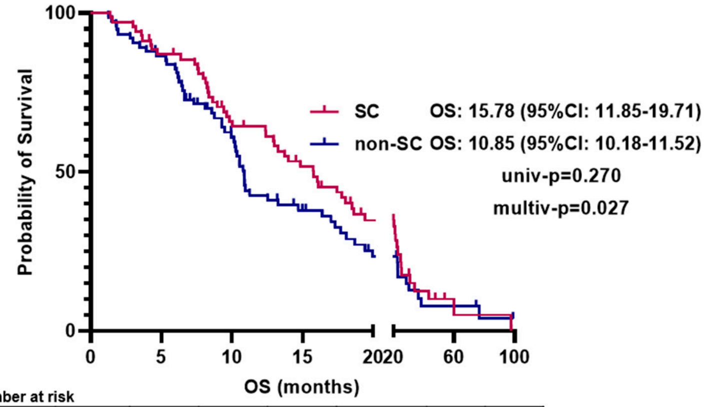

Number at risk

months	0	5	10	15	20	60	99.1
SC	69	59	43	32	18	1	0
non-SC	74	63	40	22	12	3	1

Supplementary Figure S2. Kaplan-Meier curves for progression free survival (PFS) and overall survival (OS)according to the morphological subtype in patients with advanced extra-pulmonary neuroendocrine carcinoma who received at least one line of palliative chemotherapy alone or in combination with radiotherapy (*n*=144). PFS and OS shown in the graphs refer to median values calculated by Kaplan-Meier analysis. Univ-p = p value in the univariable Cox-regression analysis. Multiv-p = p value in the multivariable Cox-regression analysis.

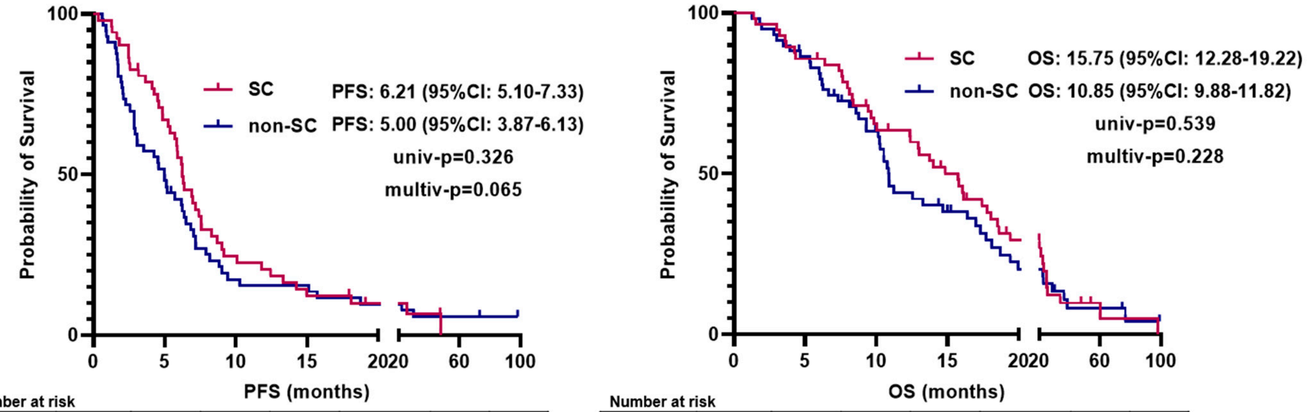

Number at risk

months	0	5	10	15	20	60	98.1
SC	52	34	12	6	3	0	0
non-SC	57	28	9	8	5	2	1

Number at risk

months	0	5	10	15	20	60	99.1
SC	56	48	35	25	13	1	0
non-SC	59	50	33	18	9	3	1

Supplementary Figure S3. Kaplan-Meier curves for progression free survival and overall surviva-laccording to the morphological subtype in patients with advanced extra-pulmonary neuroendocrine carcinoma who received first-line palliative platinum/etoposide alone or in combination with radiotherapy (*n*=116). PFS and OS shown in the graphs refer to median values calculated by Kaplan-Meier analysis. Univ-p = p value in the univariable Cox-regression analysis. Multiv-p = p value in the multivariable Cox-regression analysis.
